# Chemical Composition and Neuroprotective Properties of Indonesian Stingless Bee (*Geniotrigona thoracica*) Propolis Extract in an In-Vivo Model of Intracerebral Hemorrhage (ICH)

**DOI:** 10.3390/nu16121880

**Published:** 2024-06-14

**Authors:** Steven Tandean, Iskandar Japardi, Muhammad Rusda, Rr Suzy Indharty, Aznan Lelo, Renindra Ananda Aman, Mustafa Mahmud Amin, Andre Marolop Pangihutan Siahaan, Putri Chairani Eyanoer, Celine Augla D’Prinzessin, Ronny Lesmana, Milena Popova, Boryana Trusheva, Vassya Bankova, Felix Zulhendri

**Affiliations:** 1 Philosophy Doctor in Medicine Programme, Faculty of Medicine, Universitas Sumatera Utara, Medan 20155, Indonesia; steven.tandean@usu.ac.id (S.T.); m.rusda@usu.ac.id (M.R.); rrsuzyi@yahoo.com (R.S.I.); aznanlelo@yahoo.com (A.L.); mustafa.mahmud@usu.ac.id (M.M.A.); andremarolop@usu.ac.id (A.M.P.S.); putrieyanoer@usu.ac.id (P.C.E.); 2 Department of Neurosurgery, Faculty of Medicine, Universitas Sumatera Utara, Medan 20155, Indonesia; 3 Division of Reproductive Endocrinology and Infertility, Department of Obstetrics and Gynaecology, Faculty of Medicine, Universitas Sumatera Utara, Medan 20155, Indonesia; 4 Department of Pharmacology and Therapeutics, Faculty of Medicine, Universitas Sumatera Utara, Medan 20155, Indonesia; 5 Department of Neurosurgery, Faculty of Medicine, Universitas Indonesia, Cipto Mangunkusumo National General Hospital, Jakarta 10430, Indonesia; rajohnwien@gmail.com; 6 Department of Psychiatry, Faculty of Medicine, Universitas Sumatera Utara, Medan 20155, Indonesia; 7 Department of Community and Preventive Medicine, Faculty of Medicine, Universitas Sumatera Utara, Medan 20155, Indonesia; 8 Undergraduate Program in Medicine, Faculty of Medicine, Universitas Sumatera Utara, Medan 20155, Indonesia; auglaceline87@gmail.com; 9 Physiology Division, Department of Biomedical Sciences, Faculty of Medicine, Universitas Padjadjaran, Kabupaten Sumedang, West Java 45363, Indonesia; ronny@unpad.ac.id; 10 Institute of Organic Chemistry with Centre of Phytochemistry, Bulgarian Academy of Sciences, 1113 Sofia, Bulgaria; milena.popova@orgchm.bas.bg (M.P.); boryana.trusheva@orgchm.bas.bg (B.T.); vassya.bankova@orgchm.bas.bg (V.B.); 11 Department of Pharmacology and Clinical Pharmacy, Faculty of Pharmacy, Universitas Padjadjaran, Kabupaten Sumedang, Jawa Barat 45363, Indonesia; 12 Kebun Efi, Kabupaten Karo, Sumatera Utara 22171, Indonesia

**Keywords:** propolis, stroke, intracerebral hemorrhage, functional food, neuro-protective

## Abstract

Stroke is the world’s second-leading cause of death. Current treatments for cerebral edema following intracerebral hemorrhage (ICH) mainly involve hyperosmolar fluids, but this approach is often inadequate. Propolis, known for its various beneficial properties, especially antioxidant and anti-inflammatory properties, could potentially act as an adjunctive therapy and help alleviate stroke-associated injuries. The chemical composition of *Geniotrigona thoracica* propolis extract was analyzed by GC-MS after derivatization for its total phenolic and total flavonoid content. The total phenolic content and total flavonoid content of the propolis extract were 1037.31 ± 24.10 μg GAE/mL and 374.02 ± 3.36 μg QE/mL, respectively. By GC-MS analysis, its major constituents were found to be triterpenoids (22.4% of TIC). Minor compounds, such as phenolic lipids (6.7% of TIC, GC-MS) and diterpenic acids (2.3% of TIC, GC-MS), were also found. Ninety-six Sprague Dawley rats were divided into six groups; namely, the control group, the ICH group, and four ICH groups that received the following therapies: mannitol, propolis extract (daily oral propolis administration after the ICH induction), propolis-M (propolis and mannitol), and propolis-B+A (daily oral propolis administration 7 days prior to and 72 h after the ICH induction). Neurocognitive functions of the rats were analyzed using the rotarod challenge and Morris water maze. In addition, the expression of NF-κB, SUR1-TRPM4, MMP-9, and Aquaporin-4 was analyzed using immunohistochemical methods. A TUNEL assay was used to assess the percentage of apoptotic cells. Mannitol significantly improved cognitive–motor functions in the ICH group, evidenced by improved rotarod and Morris water maze completion times, and lowered SUR-1 and Aquaporin-4 levels. It also significantly decreased cerebral edema by day 3. Similarly, propolis treatments (propolis-A and propolis-B+A) showed comparable improvements in these tests and reduced edema. Moreover, combining propolis with mannitol (propolis-M) further enhanced these effects, particularly in reducing edema and the Virchow-Robin space. These findings highlight the potential of propolis from the Indonesian stingless bee, *Geniotrigona thoracica*, from the Central Tapanuli region as a neuroprotective, adjunctive therapy.

## 1. Introduction

In 2019, the global prevalence of stroke affected 101.5 million individuals. Among these cases, ischemic stroke was the most common, impacting 77.2 million people. Intracerebral hemorrhage affected 20.7 million individuals, while subarachnoid hemorrhage accounted for 8.4 million cases [1]. A hemorrhagic stroke is a type of stroke that occurs when a blood vessel in the brain ruptures and bleeds. This bleeding causes damage to brain cells and tissues due to the increased pressure and reduced blood flow in the affected area [2]. Over the past two decades, significant advances have been made in understanding hemorrhagic stroke, particularly in terms of pathological mechanisms and novel treatments. However, many questions regarding treatment efficacy in improving outcomes after hemorrhagic stroke, especially intracerebral hemorrhage (ICH), remain unanswered. Consequently, hemorrhagic stroke continues to be a major cause of mortality and permanent disability. A key focus of preclinical research on ICH is the development of therapies that can be translated into clinical practice to limit occurrence and expansion, reduce injury severity, and enhance repair and functional recovery [3].

Hemorrhagic stroke typically occurs spontaneously, with symptoms worsening rapidly within minutes to hours. The resulting neurological deficits depend on the location and volume of the hematoma and the subsequent cerebral edema [4,5]. Approximately 30% of cases experience hematoma expansion within the first 24 h. The mass effect of primary bleeding can cause lesions to migrate and penetrate into less dense white matter and ventricles, leading to increased intracranial pressure [5]. Perihematomal edema (PHE) and damage to local neurons typically develop within the first 24 h and progressively worsen over 14 days post-onset [2,4,6,7,8].

In the acute phase of ICH, the hypertonic dehydration method, using agents like mannitol and hypertonic saline, is commonly employed to decrease intracranial pressure [8]. Mannitol injection via the internal carotid artery in animal models has been shown to increase blood–brain barrier (BBB) permeability by 4–5 times, with a reversal of this permeability occurring within minutes. This effect is thought to be due to mannitol’s ability to induce shrinkage of cerebrovascular endothelial cells and vasodilation. However, if not administered carefully and monitored closely, mannitol can accumulate in brain lesions, increasing osmotic pressure and potentially causing water from PHE to flow into the hematoma, leading to hematoma expansion and possible midline shift [8,9]. Despite hyperosmolar fluids being the preferred therapy for brain edema, the Brain Trauma Foundation (2017) noted a lack of concrete evidence supporting the clinical outcomes of specific hyperosmolar agents in brain injury cases [10].

Propolis, a glue-like product collected by honeybees from leaf buds and resins, has been used since ancient times for various ailments. Its biological activities, derived from constituents like phenolic acids, flavonoids, and terpenoids, include antioxidant, anti-inflammatory, antimicrobial, immunomodulatory, anesthetic, anti-tumor, anti-ulcer, neuroprotective, and wound healing effects [11,12,13]. Additionally, propolis can prevent the loss of dopaminergic neurons induced by 6-hydroxydopamine through mitochondrial permeability transition inhibition and can reduce the expression of proinflammatory factors in human neuronal–glial cells via NF-κb inhibition [14,15]. Given the ongoing high mortality and morbidity rates, our aim is to investigate whether adding stingless bee propolis to current hypertonic dehydration therapy, particularly mannitol, can effectively reduce brain edema following ICH.

## 2. Materials and Methods

### 2.1. Propolis Extract Preparation

Two hundred grams of raw propolis, harvested from the hives of *Geniotrigona thoracica*, were initially physically assessed and cleaned to remove larger contaminants. The cleaned propolis was then crushed with a mortar and pestle to a sand-like consistency and mixed with 1 L of 100% glyceric solution. The extraction was carried out in a 2-L beaker on a hot plate set to 95 °C, with the mixture stirred constantly for 12 h. The mixture was then left for another 12 h to allow the solid phase of the propolis to separate and be removed manually. The solution was then passed through Whatman No. 1 cellulose filter paper repeatedly until a relatively clean solution was obtained.

### 2.2. Total Phenolics and Flavonoids of the Propolis Extract

Total phenolic and flavonoid measurements were obtained in triplicates. The blank sample was 100% glycerol. The determination of the total phenolic content in the propolis extract was conducted using the Folin–Ciocalteu method, with values expressed as gallic acid equivalents (concentration range 100–500 μg/mL) [16]. Initially, 1 mL of the extract solution was combined with 2.5 mL of 10% (*w*/*v*) Folin–Ciocalteu reagent. This mixture was left to incubate for 5 min, after which 2 mL of a 75% Na_2_CO_3_ solution was added. The mixture was then incubated at 50 °C for 10 min, with periodic stirring. Following this, the sample was cooled, and its absorbance was measured at 765 nm using a UV-Vis spectrophotometer (Analytik Jena Specord 200, Analytik Jena AG, Jena, Germany).

A standard curve was prepared using gallic acid solutions at concentrations ranging from 100 to 500 μg/mL. Each standard solution was treated in the same manner as the propolis extract, by combining 1 mL of the standard solution with 2.5 mL of 10% (*w*/*v*) Folin–Ciocalteu reagent, incubating for 5 min, adding 2 mL of a 75% Na_2_CO_3_ solution, and incubating at 50 °C for 10 min with periodic stirring. After cooling, the absorbance of each standard solution was measured at 765 nm using the same UV-Vis spectrophotometer. The absorbance values obtained were used to construct a standard curve by plotting absorbance against gallic acid concentration. The total phenolic content of the propolis extract was then determined by comparing its absorbance to the standard curve.

For the total flavonoid content, the aluminum chloride colorimetric method was used [16]. A diluted methanolic solution of propolis extract (10 mL) was mixed with 3 mL of 5% (*w*/*v*) NaNO_2_ solution and 40 mL of distilled water. After a 5-min reaction period, 3 mL of 10% (*w*/*v*) aluminum chloride solution was added. Following another 5-min interval, 20 mL of NaOH solution was incorporated. The mixture’s absorbance was measured at 415 nm after a 15-min incubation at room temperature. The results were reported as quercetin equivalents (mM QE/mL).

To prepare the standard curve, quercetin solutions at concentrations ranging from 100 to 500 μg/mL were used. Each standard solution was treated in the same manner as the propolis extract, by mixing 10 mL of the standard solution with 3 mL of 5% (*w*/*v*) NaNO_2_ solution and 40 mL of distilled water. After a 5-min reaction period, 3 mL of 10% (*w*/*v*) aluminum chloride solution was added. Following another 5-min interval, 20 mL of NaOH solution was incorporated. The mixture was then incubated for 15 min at room temperature before measuring the absorbance at 415 nm using a UV-Vis spectrophotometer (Analytic Jena Specord 200, Germany). The absorbance values obtained from these standard solutions were plotted against their respective quercetin concentrations to construct the standard curve. The total flavonoid content of the propolis extract was determined by comparing its absorbance to the standard curve.

### 2.3. GC-MS Analyses of the Glyceric Extract of Propolis

A small part of the glyceric extract was diluted with water and extracted with ethyl acetate in order to remove the glycerol. The ethyl acetate extract was evaporated to dryness and derivatized; about 5 mg of dry extract was mixed with 50 μL dry pyridine and 75 μL N,O-bis(trimethylsilyl) trifluoracetamide (Sigma-Aldrich, Darmstadt, Germany), was heated at 80 °C for 20 min, and analyzed by GC-MS.

The tandem mass spectrometer, GC-MS-TQ 8050 NX Shimadzu (Tokyo, Japan), was used for the GC-MS analysis, operating in the Q3 scan mode and equipped with a 30 m long, 250 μm i.d., and 0.25 μm film thickness SAPIENS-5MS capillary column (Teknokroma, Barcelona, Spain). The oven temperature program involved an increase from 100° to 300 °C at a rate of 5 °C/min, with a 25 min hold at 300 °C Carrier gas He, at a constant flow rate of 0.8 mL/min. The pressure was 45.9 kPa, split ratio was 75:1, injector temperature was 280 °C, interface temperature was 310 °C, and ionization voltage was 70 eV. The obtained GC-MS data were analyzed using AMDIS software (version 2.73), which allows deconvolution of overlapping mass spectra yielding mass spectra of higher “purity” for very small chromatographic peaks. Compounds were identified primarily based on their MS fragmentation and were further confirmed by comparison with spectra of authentic samples and matching with the literature and commercial library data (NIST, Wiley). The percentage values in Table 1 refer to the percentage of the Total Ion Current (TIC) and are semi-quantitative (semi-quantification was performed by internal normalization).

### 2.4. In-Vivo Study

This study was conducted at the iRATco Laboratory in Bogor. All experimental procedures complied with the regulations of the Ethical Committee of the Faculty of Medicine at Universitas Sumatera Utara, registered under article number 39/KEPK/USU/2023. We employed a post-test-only control group design. Ninety-six Sprague Dawley rats (from Federer formula) were divided into six groups; namely, the untreated control group (negative control), the ICH group (positive control), and four ICH groups that received the following therapies: mannitol, propolis (daily oral propolis administration after the ICH induction), propolis-M (propolis and mannitol), and propolis-B+A (daily oral propolis administration 7 days prior to and after the ICH induction). The animals were tested for neuro-motoric performance and then sacrificed for histochemical analyses on day 3 (6 animals), day 7 (6 animals), and day 14 (4 animals). Prior to the experiment, the animals were acclimatized for 7 days to restore their physiological systems. They were placed in square cages measuring 30 cm × 40 cm × 15 cm, with husk pads that were cleaned every 3 days or when dirty. The temperature was maintained at 22–24 °C, with a 12-h light and dark cycle. They were fed 15–20% of their body weight daily. The animals were treated the same way after the experiment. Inflammation and apoptosis reached their peak 24–48 h after injury and started to decline on the 3rd day. The apoptosis process ceased on the 7th day and was followed by the repair and regeneration process [17]. All animals were treated according to the principles of the 3Rs (Reduction, Replacement, and Refinement) and the 5Fs (Freedom from hunger and thirst; Freedom from discomfort; Freedom from pain, injury, or disease; Freedom to express normal behavior; and Freedom from fear and distress) [18].

#### 2.4.1. The ICH Model

We induced ICH using the Deinsberger method, where a double injection of autologous blood was administered on the right hemisphere of the brain [19]. The animals were placed in a prone position and secured on a stereotactic head frame. A burr-hole was made 3.5 mm laterally and 1 mm anterior to the coronal suture. A 26 G polyethylene cannula with a mandrin was inserted through the dural opening into the caudate nucleus at a depth of 7 mm. Fresh autologous blood was drawn from an arterial catheter into the polyethylene catheter. First, 15 μL of blood was injected at a rate of 10 μL/min. After a 7-min wait, allowing the initial injection to form a clot, a second injection of 35 μL of blood was administered at the same rate. This method has been applied in several studies using small animals. The superiority of this method is that it avoids blood flowing back into the subarachnoid space [19]. The caudate nucleus is targeted for lesioning due to its critical function in various higher neurological functions. The caudate nucleus plays a role in the execution of movement plans, learning, memory, reward, motivation, emotion, romantic interaction, and associative learning [20].

The control group received no intervention. In the ICH group, ICH was induced, followed by no therapeutic treatment. The propolis group animals received propolis diluted in 2 mL of water orally for 72 h post-ICH at a dose of 31 μL/kg BW/day. The propolis dose used was based on our preliminary data on the efficacy of the hydroglyceric extract in alleviating acute respiratory distress syndrome (ARDS) in an in-vivo model (unpublished data). The mannitol group received 20% (*v*/*v*) mannitol intraperitoneally at 5 mL/kg twice daily. The propolis-M group received a combination of propolis and mannitol at the same dosages. In the propolis-B+A group, animals were administered propolis at the same daily dose for 7 days prior to the ICH induction and daily for 3 days after the ICH induction.

#### 2.4.2. Assessment of Cognitive–Motor Functions

The behavioral assessments were carried out in accordance to [21]. Motor function was analyzed using a rotarod test. Briefly, a rod measuring 3 cm in diameter and 40 cm in length is wrapped in adhesive plasters to enhance its roughness. This rod is then rotated by an electric motor at a speed of 4 revolutions per minute (rpm). Below the rod, a landing platform with a soft surface is positioned 18 cm underneath to safeguard animals in case they fall. Prior to testing, the animals undergo training to acclimate to the task. Initially, they are placed on the stationary rod for 30 s, followed by exposure to a slow rotation of 4 rpm. Training continues until the animals can remain on the rotating rod for a minimum of one minute. In the testing trial, the animals are placed on the rod as it gradually accelerates from 4 rpm to 40 rpm over a 300-s period. The time taken before they fall onto the platform is recorded. Rodents with compromised locomotor abilities tend to fall off the rod more quickly than those with normal function.

The Morris Water Maze test assessed spatial learning and memory recall abilities. The animals are introduced into a large circular pool measuring 180 cm in diameter. The pool, divided into four quadrants, contains a platform concealed just beneath the water’s surface in one of the quadrants. To obscure the platform, the water is tinted with paint. Animals can escape by locating this hidden platform. The time taken to find the platform serves as an indicator of cognitive function, where increased latency suggests memory impairments and cognitive decline. Additionally, the duration spent in the quadrant containing the platform can be a valuable measure. With advanced video tracking software, the path taken by the animals, their total swimming distance, and average swimming speed are tracked, visualized, and analyzed.

#### 2.4.3. Cerebral Edema Analyses

One of the harmful pathological states that occurs after stroke and brain injury is brain edema. This excessive accumulation of extracellular fluid induces an increase in intracranial pressure and causes permanent damage to nerve function [22]. After the cognitive–motoric tests, the animals were sedated with ether and subsequently decapitated. Sixty minutes prior to euthanasia, 2% Evans Blue at a dosage of 4 mL/kg in normal saline was injected into the caudal vein. The cerebrum was sectioned coronally into three segments, embedded in paraffin blocks, and sliced into sections 4 μm thick. The first segment, located directly above the injection site, was utilized to identify the ipsilateral and contralateral parts of the Intracerebral Hemorrhage (ICH). These parts were then dried for 72 h at 110 °C to ascertain the dry weight of each hemisphere. The cerebral edema index was calculated using the formula:

$$Edema\;Index\;\left(\%\right)=\frac{weight\;of\;right\;hemisphere\;\left(g\right)-weight\;of\;left\;hemisphere\;(g)}{weight\;of\;left\;hemisphere\;(g)}\times 100\%$$

Cerebral edema was further evaluated by measuring the Virchow-Robin spaces (μm). The remaining segments, on the side ipsilateral to the ICH, were used in immunohistochemical analyses to investigate the proteins associated with edema and apoptosis.

#### 2.4.4. Immunohistochemical Analyses

The expression of NF-κb, SUR1-TRPM4, MMP-9, and Aquaporin-4 was analyzed using immunohistochemical methods adapted from [23]. The primary antibodies used were NF-κb (Abcam, Cambridge, UK), SUR-1-TRPM4 (Elabscience, Houston, TX, USA), MMP-9 (Elabscience, Houston, TX, USA), and Aquaporin-4 (Bioss, Woburn, MA, USA). The samples were rinsed with PBS pH 7.4, then blocked with 3% H_2_O_2_ for 20 min. After three more PBS washes, unspecific proteins were blocked using 5% FBS (0.25% Triton X-100). Samples were incubated with a primary antibody for 60 min, then with an anti-rabbit HRP conjugate (Abcam, Cambridge, UK) for 40 min, with PBS washes in between. After Diamino Benzidine (DAB) treatment and counterstaining with Mayer Hematoxylin, samples were analyzed using light microscopy. Image analysis was conducted on five different fields at 400× magnification using an Olympus BX40 light microscope, equipped with an Olympus DP71 camera. The camera’s resolution was 2040 × 1536 pixels, and the images were saved in TIFF format.

The initial step in the analysis involved color deconvolution using the IHC Profiler plugin. Subsequently, the intensity of immunostaining in cells was quantified in the deconvoluted DAB images. The average staining intensities of all measured cells across ten high-power fields of view were calculated for each sample. For quantification, ImageJ software (version 1.53g) was used. Images were first loaded into ImageJ and converted to 8-bit grayscale. Color deconvolution was performed using the IHC Profiler plugin, selecting the DAB-Hematoxylin option to separate the DAB (brown) staining from the Hematoxylin (blue) staining. Threshold settings were adjusted using the Threshold function (Image > Adjust > Threshold), using the Otsu method, to isolate positive staining. A binary image was created by applying the threshold, typically set within a 0–255 range. The particle analysis was then conducted (Analyze > Analyze Particles), with size set to 50-Infinity pixels and circularity to 0.20–1.00 to filter likely positive staining cells. Options Show > Outlines and Display Results were selected to visualize and record identified particles.

The intensity of immunostaining in cells was quantified by measuring the area, mean gray value, and integrated density for each positive cell. The average staining intensities of all measured cells across ten high-power fields of view were calculated for each sample. Staining intensities were categorized as positive or negative based on established thresholds, and the expression area per field of view was quantified and expressed as a percentage using the formula:

$$Percentage\;of\;cells\;\left(\%\right)=\frac{Number\;of\;cells\;expressing\;the\;biomarker}{Total\;number\;of\;cells\;}\times 100\%$$

#### 2.4.5. TUNEL Assay

In addition, we also carried out a TUNEL assay to determine the percentage of apoptotic cells in all groups, including the control groups. This method was carried out according to instructions provided by the kit manufacturer (Elabscience, Houston, TX, USA). Briefly, the samples were stripped of paraffin wax using xylene, followed by rehydration through gradual ethanol treatment, and then soaked in a 0.1 M PBS solution for 15 min. Subsequently, they were exposed to proteinase K at a concentration of 20 μg/mL for 20 min at room temperature. The sections were then subjected to a treatment involving a mixture of 3% hydrogen peroxide (H_2_O_2_) in methanol for 20 min, which served to deactivate the naturally occurring peroxidase. After a 15-min PBS wash, the sections were placed in a labeling reaction mixture that contained terminal deoxynucleotidyl transferase and deoxynucleotides, left overnight at a low temperature of 4 °C. Following the overnight incubation, all sections were rinsed with PBS for 15 min and then exposed to horseradish peroxidase at a dilution of 1:500 for 30 min at room temperature. Subsequently, the sections underwent an extensive PBS wash for 15 min and were treated with a DAB solution, consisting of 30 mg of DAB and 200 μL of H_2_O_2_ per 100 mL of PBS, for 10 min at room temperature in a dark environment. After rinsing the sections under running water, they were all briefly counterstained with H&E (hematoxylin and eosin) for 30 s. Finally, the sections went through a dehydration process, incrementally with ethanol concentrations, were cleared with xylene, and were enclosed with a cover slip. The TUNEL staining method allowed for the identification of apoptotic nuclei based on the presence of dark brown staining within the sections. ImageJ software quantified the apoptotic cells per ten high-power fields of view, which were expressed as a percentage of apoptotic cells according to the formula as follows:

$$Percentage\;of\;apoptotic\;cells\;\left(\%\right)=\frac{Number\;of\;apoptotic\;cells\;}{Total\;number\;of\;cells\;}\times 100\%$$

Briefly, images were saved in TIFF format to prevent quality loss. In ImageJ, images were converted to 8-bit grayscale (Image > Type > 8-bit). The threshold function was used (Image > Adjust > Threshold) to isolate positive staining, employing the Otsu method within a 0–255 range. The binary image created from this thresholding process was then analyzed using the Analyze Particles function (Analyze > Analyze Particles), with size and circularity parameters set to 50-Infinity pixels and 0.20–1.00, respectively, to filter particles representing apoptotic nuclei. The options to Show > Outlines and Display Results were selected to visualize and record identified particles. Apoptotic nuclei were distinguished based on morphological features, such as size, shape, and staining intensity, with only clear, round, strongly stained particles counted as positive cells. The analysis was performed on ten high-power fields of view per sample to account for variability, and both positive and negative controls were included in each analysis batch to ensure specificity and sensitivity of the TUNEL staining.

### 2.5. Statistical Analysis

A Shapiro-Wilk test confirmed that the data were not normally distributed. A pairwise correlation analysis was performed using the Kruskal-Wallis One-Way Analysis of Variance. Data are presented as quartiles for all rats in all groups. The Kruskal-Wallis One-Way Analysis of Variance was used to compare treatment groups, followed by a post-hoc Dunn’s test to identify specific group differences. Statistical analysis was conducted using IBM SPSS Statistics (version 29.0.0.0), with *p* < 0.05 considered statistically significant.

## 3. Results

The total phenolic content and total flavonoid content in the propolis extract were 1037.31 ± 24.10 μg GAE/mL and 374.02 ± 3.36 μg QE/mL, respectively. The results of the GC-MS analysis of the extract are presented in Table 1. The chemical profiling of the propolis extract showed that the major constituents were triterpenoids, with cycloartenol being the main component. Phenolic lipids, such as cardanols (alk(en)yl phenols), cardols (alk(en)yl resorcinols), and anacardic acids, were also identified.

**Figure 1:**
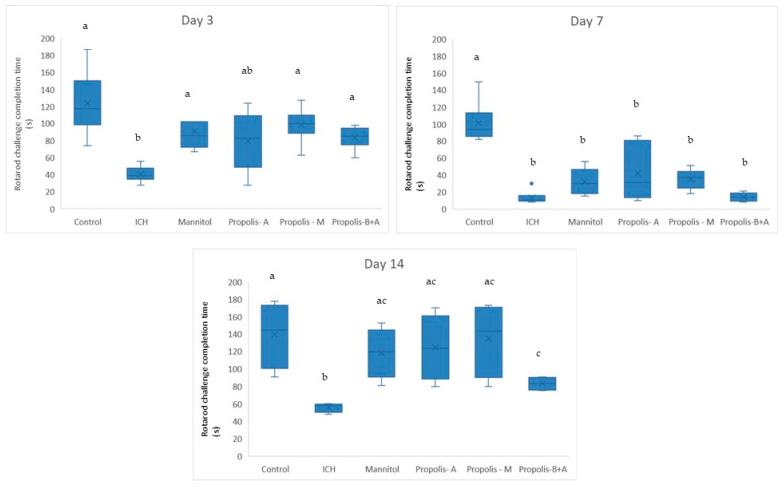
Completion time (in s) for the Rotarod challenge on days 3, 7, and 14. Different letters indicate a statistically significant difference (*p* < 0.05). For example, groups labeled with ‘a’ and ‘b’ are significantly different from each other, while groups labeled with ‘ab’ and ‘b’ are not significantly different from each other.

Figure 1 displays the completion times for the rotarod challenge. On day 3, there was no significant difference in rotarod performance among the control, mannitol, propolis-M, and propolis-B+A groups (*p* > 0.05). These groups performed better compared to the ICH group (*p* < 0.05). However, the propolis-A group did not show a significant difference from the ICH group (*p* > 0.05). On day 7, animals in the treatment groups performed significantly worse compared to the control group (*p* < 0.05). No significant difference was observed among the treatment groups (*p* > 0.05). By day 14, the animals in the treatment groups had improved performance compared to day 7. There was a significant difference between the ICH group and all other groups (*p* < 0.05), with all treatment groups performing better than the ICH group. There was no significant difference between the control, mannitol, propolis-A, and propolis-M groups (*p* > 0.05). However, the propolis-B+A group showed worse performance compared to the control and propolis-M groups (*p* < 0.05).

**Figure 2:**
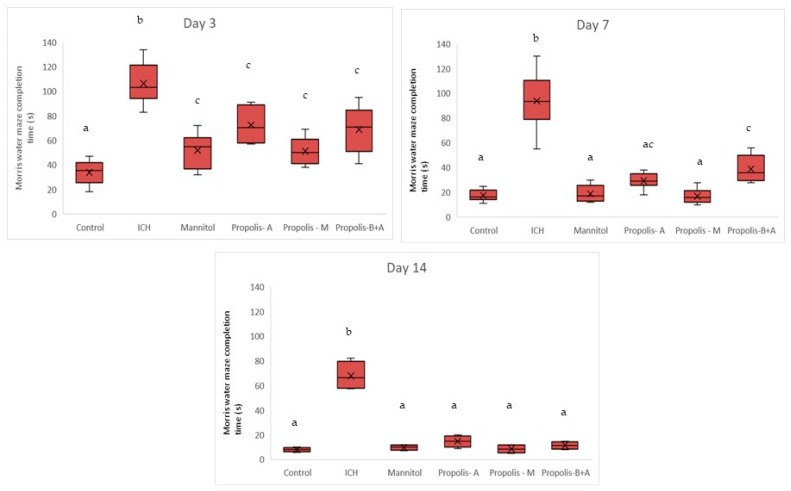
Morris water maze completion time (s) on days 3, 7, and 14. Different letters indicate a statistically significant difference (*p* < 0.05). For example, groups labeled with ‘a’ and ‘b’ are significantly different from each other, while groups labeled with ‘ab’ and ‘b’ are not significantly different from each other.

Figure 2 shows the completion times for the Morris water maze. On day 3, the ICH group had the longest time compared to all other groups (*p* < 0.05). There was no significant difference between the control, mannitol, and propolis-M groups (*p* > 0.05). The propolis-A and propolis-B+A groups had shorter times (indicating better performance) compared to the ICH group, but not to the level of the control group (*p* < 0.05). However, the performance of the propolis-A and propolis-B+A groups was similar to that of the mannitol and propolis-M groups (*p* > 0.05). A similar trend was observed on day 7, with all animals in the treated groups, except for the ICH group, performing better compared to day 3. There was no significant difference between the control group and the mannitol, propolis-A, and propolis-M groups (*p* < 0.05). However, there was a significant difference between the control and the propolis-B+A group (*p* < 0.05). On day 14, all treated animals appeared to perform similarly to the control group (*p* > 0.05). Moreover, there was still a significant difference between the ICH group and all other groups (*p* < 0.05).

**Figure 3:**
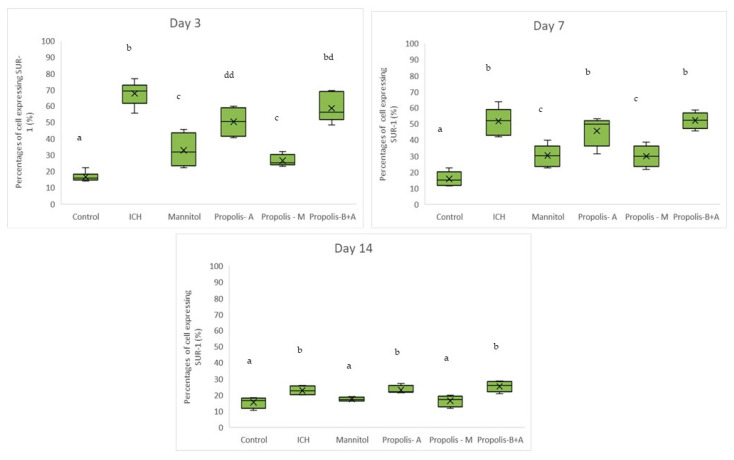
Percentage of cells expressing SUR-1 on days 3, 7, and 14. Different letters indicate a statistically significant difference (*p* < 0.05). For example, groups labeled with ‘a’ and ‘b’ are significantly different from each other, while groups labeled with ‘ab’ and ‘b’ are not significantly different from each other.

Figure 3 displays the percentages of cells expressing SUR-1. The control group had the lowest percentage of cells expressing SUR-1 (*p* < 0.05) compared to all other groups. The mannitol and propolis-M groups had lower percentages compared to the ICH, propolis-A, and propolis-B+A groups (*p* < 0.05). Additionally, there was no significant difference in SUR-1 expression between the propolis-A and mannitol groups (*p* > 0.05). A similar trend was observed on day 7. By day 14, there was no significant difference between the control group and both the mannitol and propolis-M groups (*p* > 0.05). Furthermore, there was no significant difference between the ICH group and both the propolis-A and propolis-B+A groups (*p* > 0.05).

**Figure 4:**
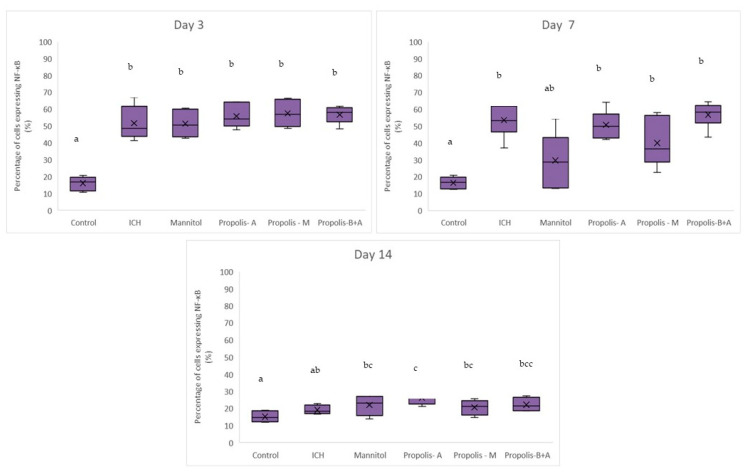
Percentage of cells expressing NF-κB on days 3, 7, and 14. Different letters indicate a statistically significant difference (*p* < 0.05). For example, groups labeled with ‘a’ and ‘b’ are significantly different from each other, while groups labeled with ‘ab’ and ‘b’ are not significantly different from each other.

**Figure 5:**
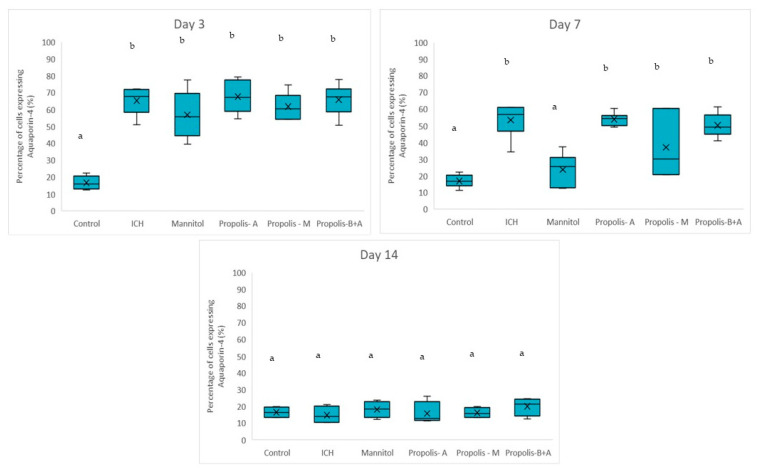
Percentage of cells expressing Aquaporin-4 on days 3, 7, and 14. Different letters indicate a statistically significant difference (*p* < 0.05). For example, groups labeled with ‘a’ and ‘b’ are significantly different from each other, while groups labeled with ‘ab’ and ‘b’ are not significantly different from each other.

Figure 4 shows the percentages of cells expressing NF-κB. No significant difference was observed among all other groups compared to the control group in terms of the percentage of cells expressing NF-κB on day 3. On day 7, there was a difference between the control group and the ICH, propolis-A, propolis-M, and propolisA+B groups (*p* < 0.05). By day 14, the control group was not significantly different from the ICH group (*p* > 0.05). However, all other treatment groups had slightly higher NF-κB expression compared to the control group (*p* < 0.05). Additionally, Figure 5 showed the percentage of cells expressing Aquaporin-4. There was no significant difference between the treatment groups and the ICH group (*p* > 0.05). Conversely, there was a significant difference between the control group and all other groups (*p* < 0.05). On day 7, there was a significant difference between the mannitol group and the ICH group (*p* < 0.05). However, there was no significant difference between the ICH group and the other treatment groups (*p* > 0.05). On day 14, all groups had a similar level of cells expressing Aquaporin-4 (*p* > 0.05).

**Figure 6:**
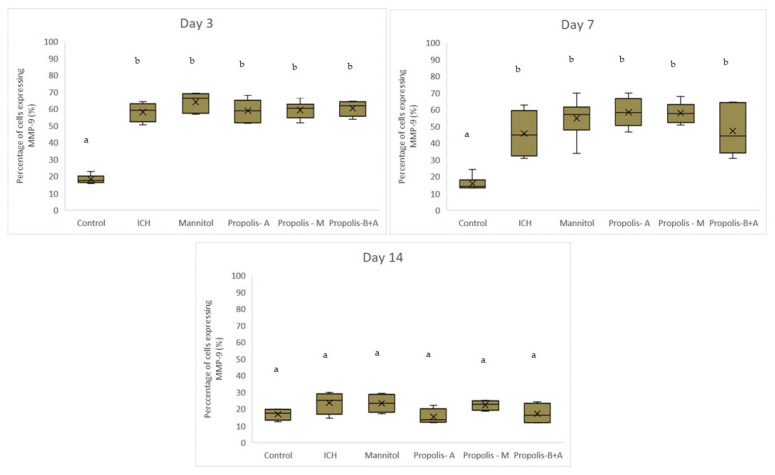
Percentage of cells expressing MMP-9 on days 3, 7, and 14. Different letters indicate a statistically. For example, groups labeled with ‘a’ and ‘b’ are significantly different from each other, while groups labeled with ‘ab’ and ‘b’ are not significantly different from each other.

**Figure 7:**
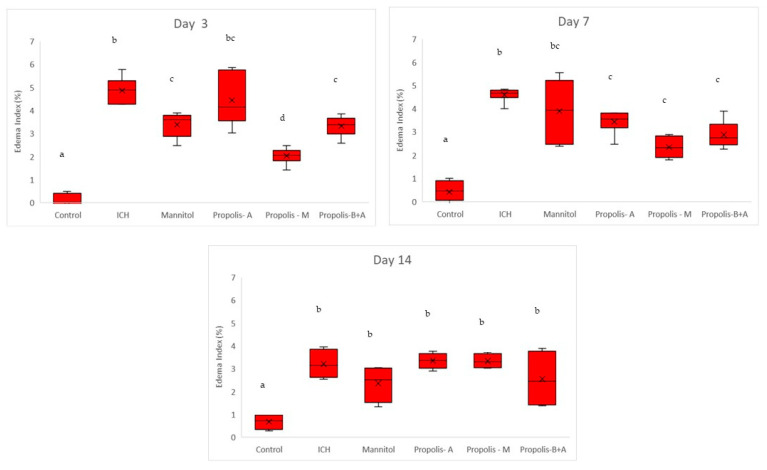
Edema index (%). Different letters indicate a statistically significant difference (*p* < 0.05). For example, groups labeled with ‘a’ and ‘b’ are significantly different from each other, while groups labeled with ‘ab’ and ‘b’ are not significantly different from each other.

Figure 6 shows the percentage of cells expressing MMP-9. There was no significant difference between the treatment groups and the ICH group (*p* > 0.05). Conversely, there was a significant difference between the control group and all other groups (*p* < 0.05). A similar trend was observed on day 7. By day 14, there was no difference among all groups (*p* > 0.05). Moreover, Figure 7 shows the edema index. As expected, the control group had the lowest edema index on days 3, 7, and 14. On day 3, the edema indices in the mannitol, propolis-M, and propolis-B+A groups were significantly lower compared to the ICH group, with the lowest index recorded in the propolis-M group (*p* < 0.05). A similar trend was observed on day 7. By day 14, there was no difference among all groups (except for the control group).

**Figure 8:**
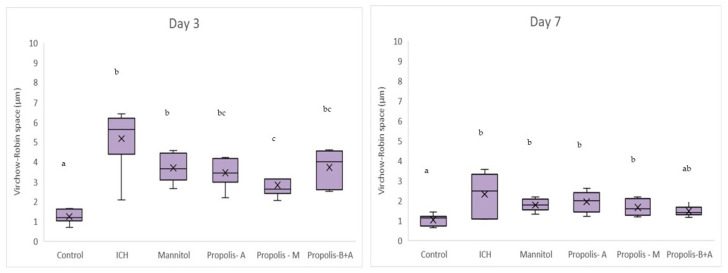
Virchow-Robin space (μm). Different letters indicate a statistically significant difference (*p* < 0.05). For example, groups labeled with ‘a’ and ‘b’ are significantly different from each other, while groups labeled with ‘ab’ and ‘b’ are not significantly different from each other.

**Figure 9:**
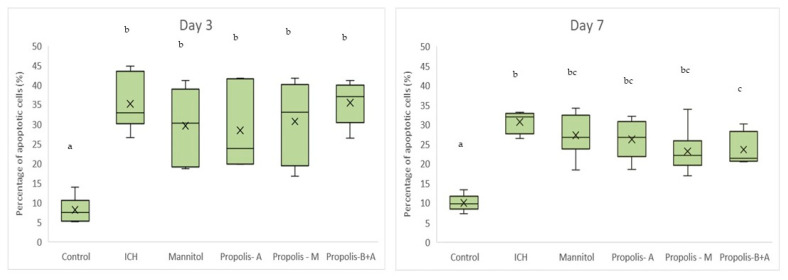
Percentage of apoptotic cells based on the TUNEL assay. Different letters indicate a statistically significant difference (*p* < 0.05). For example, groups labeled with ‘a’ and ‘b’ are significantly different from each other, while groups labeled with ‘ab’ and ‘b’ are not significantly different from each other.

Figure 8 shows Virchow-Robin space (μm) measurements. On day 3, the control group had the smallest measurement, followed by the propolis-M group (*p* < 0.05). The ICH group appeared to have the largest Virchow-Robin space; however, this did not reach statistical significance when compared to the mannitol, propolis-A, and propolis-B+A groups. On day 7, the control group had the smallest space and was not significantly different compared to the propolis-B+A group (*p* > 0.05). All other groups had similar measurements (*p* > 0.05). Furthermore, Figure 9 shows the percentage of apoptotic cells measured using the TUNEL assay. The control group had the lowest number of apoptotic cells on days 3 and 7. On day 7, the propolis-B+A group had the lowest percentage of apoptotic cells among the treatment groups and was significantly different compared to the ICH group (*p* < 0.05). Table S1 presents all the means and standard deviations of the data. 

## 4. Discussion

The data from the quantitative analyses showed that the glyceric extract of the studied sample is characterized by very low phenolic and flavonoid content compared to the poplar-type propolis, which is the most widespread honeybee propolis type in the world [24,25]. For the purpose of the GC-MS analysis, the studied glyceric extract was subjected to liquid–liquid extraction with ethyl acetate. Most of the propolis components possess low volatility and for this reason the ethyl acetate extract after evaporation to dryness was silylated employing BSTFA. The BSTFA reagent is conventional and one of the most prevalent derivatizing agents in GC and GC-MS. It is very suitable and effective for the modification of -OH and -COOH functional groups present in the molecules of propolis components to the respective trimethylsilyl ethers and trimethylsilyl esters [26]. Based on the GC-MS, the chemical profiling of the propolis extract revealed that its significant constituents were triterpenoids, with cycloartenol being the predominant one. These triterpenes were accompanied by phenolic lipids: cardanols (alk(en)yl phenols), cardols (alk(en)yl resorcinols), and anacardic acids. These compounds are taxonomic markers of the resin from the mango tree, *Mangifera indica*, a well-known and often detected source of propolis in tropical regions for both Western (*Apis mellifera*) and stingless bees [27]. In addition, a smaller amount of diterpenic acids was also identified. Diterpenes are not unusual for propolis, but have not been found in propolis from Southeast Asia and Indonesia so far. The most likely source of these diterpenic acids is the resin of the Sumatran pine, *Pinus mercusii*, as diterpenic acids are known to be its typical constituents. No other plant source available to the bees in this region can supply this type of resin. This is the first indication that *P. mercusii* can act as a resin source for propolis production, which deserves further studies. It is known that stingless bees utilize the surrounding biodiversity to gather various resins. Drescher et al. (2014) discovered that resins from several plant species target various infections and intruders of bees’ nests, working synergistically when paired [28]. Because they depend on resin for protection, bees benefit from having access to a variety of resin sources. Moreover, conifer diterpenoids have demonstrated neuroprotective activity, which may contribute to the observed effects of the studied propolis [29]. Additionally, phenolic lipids possess multifunctional activity toward the prevention of Alzheimer’s disease [30].

The protective effects of propolis in mitigating symptoms related to aneurysm, ischemia, ischemia–reperfusion, and traumatic brain injuries have been observed. Its anti-inflammatory capabilities are crucial in lessening the adverse impacts of these conditions. Propolis has been shown to decrease the levels of interleukin-6 (IL-6), TNF-α, matrix metalloproteinase-2 (MMP-2), MMP-9, monocyte chemotactic protein-1 (MCP-1), and iNOS, while it enhances the production of protective proteins, like heat shock protein-70 (hsp70) [31,32,33,34,35]. Additionally, it prevents the formation of histopathology linked to these injuries and, in some instances, fosters the growth of myelinated fibers [33,36]. Most notably, propolis significantly improves sensory–motor and other physical functions in animals that have suffered these injuries [15,18,21,22]. One significant point to note is that we administered the propolis extract orally in a dosage range that would be representative for human use. In contrast, most animal models use a much higher concentration range, typically between 5 mg and 1000 mg/kg BW. [37].

In the present study, mannitol, used as a standard treatment, showed therapeutic benefits in terms of cognitive–motor functions. This was measured using the rotarod and Morris water maze completion times, with mannitol demonstrating improvement compared to the ICH group (Figure 1 and Figure 2). Additionally, mannitol reduced the expression of SUR-1 and Aquaporin-4 (Figure 3 and Figure 5), and decreased the edema index on day 3 (Figure 7). Comparatively, treatments with propolis alone, specifically propolis-A and propolis-B+A, also resulted in significant improvements in rotarod and Morris water maze completion times compared to the ICH group (Figure 1 and Figure 2). Furthermore, the propolis-A group exhibited reduced SUR-1 expression on day 3 (Figure 3) and a lower edema index on day 7 (Figure 7). The propolis-B+A group showed a reduced edema index on both days 3 and 7 (Figure 7). Moreover, it appears that adding oral propolis treatment to mannitol (propolis-M) enhanced mannitol’s efficacy. This enhancement was evidenced by improved values in terms of the edema index on days 3 and 7 and the Virchow-Robin space on day 3 (Figure 8), compared to mannitol alone.

Brain edema plays a very important role after brain injury due to the elevation of intracranial pressure and induced neurodegeneration. Brain edema can be caused by inflammation induced by brain hematoma. MMP-9 and NF-κB are inflammation markers that play important roles in the disruption of the blood–brain barrier (BBB). Dysfunction of the BBB leads to the accumulation of fluid in the brain parenchyma. Upregulation of SUR-1 and Aquaporin-4 expression has been shown to increase blood–brain barrier permeability. One treatment strategy after brain injury is to prevent or enhance the resolution of edema. Propolis has shown potential effects by reducing inflammation and downregulating SUR-1 and Aquaporin-4 expression [32,35,38].

Recent studies have explored the efficacy of propolis in improving cognitive and motor functions, especially in aging populations and neurodegenerative conditions [39,40]. The neuroprotective role of propolis is primarily attributed to its antioxidant properties, which combat oxidative stress, a key factor in the pathogenesis of cognitive decline. Oxidative stress leads to neuronal damage and death, contributing to the deterioration of cognitive and motor functions. Propolis, with its potent antioxidant compounds, scavenges free radicals and reduces oxidative damage in neural tissues [38,41]. Furthermore, the anti-inflammatory action of propolis plays a vital role in its neuroprotective effects. Inflammation is a hallmark of several neurodegenerative diseases, and the bioactive compounds in propolis can modulate inflammatory pathways. By inhibiting the production of pro-inflammatory cytokines and reducing neuroinflammation, propolis helps in preserving neuronal integrity and function [12]. There is also evidence of propolis having neuroregenerative properties [42,43].

Clinical studies have shown that supplementation with propolis can lead to improvements in cognitive functions, as measured by standardized neuropsychological tests. These improvements are often correlated with the modulation of serum levels of inflammatory markers and other biochemical indicators of neural health. Additionally, propolis has been observed to aid in motor function, potentially benefiting individuals with motor impairments or age-related motor decline [39,40]. Moreover, the neuroprotective effects of propolis extend to its potential in enhancing synaptic plasticity and neurogenesis, processes vital for learning, memory, and motor coordination. The compounds in propolis may influence neuronal signaling pathways, supporting the maintenance and formation of synaptic connections, which are essential for cognitive and motor functions [44,45,46].

In the present study, while the propolis treatments led to a significant improvement in inflammation and apoptosis markers, predominantly observed on days 3 and 7, this alone does not seem to fully account for the significant enhancement in cognitive–motor functions observed. The suggests that the clinical improvements may be attributed to factors beyond the mere reduction of inflammation and apoptosis. We hypothesize that the observed benefits in cognitive and motor abilities are more likely linked to the antioxidant properties of propolis, a key factor in neuronal damage and cognitive decline.

Additionally, the neuroregenerative properties of propolis could play a crucial role. These properties might aid in the repair or regeneration of neural tissues, thereby contributing to the recovery of cognitive and motor functions. Propolis is also known for its neuromodulatory effects, which could influence neuronal signaling and brain function in ways that are not directly related to its anti-inflammatory and anti-apoptotic actions. Given these possibilities, it becomes evident that the therapeutic potential of propolis extends beyond its anti-inflammatory and anti-apoptotic effects. The antioxidant, neuroregenerative, and neuromodulatory properties of propolis are areas that warrant further investigation to fully understand their contribution to the observed clinical improvements.

Further in-vivo studies investigating the effects of different concentrations of propolis are warranted. These studies should aim to determine the optimal dosage and concentration that maximize the therapeutic benefits of propolis, while minimizing any potential side effects. Additionally, understanding the dose–response relationship will provide valuable insights into the mechanisms of action of propolis and its impact on various biological pathways. Such research could pave the way for the development of standardized and effective propolis-based therapies, potentially offering new treatment options for conditions such as hemorrhagic stroke. Conducting these in-vivo studies in diverse animal models will also enhance the generalizability of findings and support the eventual translation of this natural compound into clinical applications.

## 5. Conclusions

In the present study, the chemical composition of propolis produced by the Indonesian stingless bee, *Geniotrigona thoracica*, from the Central Tapanuli region was reported or the first time. The conducted chemical analyses showed that the glyceric extract of the studied sample is characterized by a relatively low phenolic and flavonoid content and a predominant terpene content. By GC-MS analysis, its major constituents were found to be triterpenoids (22.4% of TIC), with cycloartenol being the main one. Minor compounds, such as phenolic lipids (6.7% of TIC, GC-MS) and diterpenic acids (2.3% of TIC, GC-MS), were also found. The triterpenes identified, accompanied by phenolic lipids, are taxonomic markers of the resin from the mango tree, *Mangifera indica*, which determines it as the main plant source of the studied sample. The other plant source from which diterpenic acids originate is probably the resin of the Sumatran pine, *Pinus mercusii*, as diterpenic acids are known to be its typical constituents.

Further, neuroprotective properties of the glyceric propolis extract in an in-vivo model of Intracerebral Hemorrhage (ICH) were investigated. In conclusion, we observed that mannitol, a standard treatment, demonstrated therapeutic advantages in enhancing cognitive-motor functions. This improvement was evident in the decreased completion times for both the rotarod and Morris water maze tests, as seen in the ICH group (refer to Figure 1 and Figure 2). Additionally, mannitol was effective in lowering the levels of SUR-1 and Aquaporin-4. Furthermore, it significantly reduced cerebral edema, as indicated by the edema index on the third day. In comparison, treatments solely using propolis, specifically propolis-A and the combination of propolis-B+A, also yielded notable improvements. These improvements were similar to those observed with mannitol, particularly in the rotarod and Morris water maze completion times when compared to the ICH group. The group treated with propolis-A exhibited a reduction in SUR-1 expression on day 3 and a decrease in the edema index by day 7. The propolis-B+A group showed a significant reduction in the edema index on both days 3 and 7. Moreover, the study found that combining oral propolis treatment with mannitol (propolis-M) appeared to enhance mannitol’s effectiveness. This combination resulted in more pronounced improvements, particularly in reducing the edema index on days 3 and 7, and in the reduction of the Virchow-Robin space on day 3, when compared to the use of mannitol alone. These findings suggest that the adjunctive use of Indonesian stingless bee propolis extract with mannitol may offer additional therapeutic benefits in the treatment of conditions affecting cognitive–motor functions. In conclusion, Indonesian stingless bee (*G. thoracica*) propolis extract appears to be a promising functional food ingredient with neuroprotective properties illustrated by the present in-vivo study.

## Figures and Tables

**Table 1:** Compounds identified in the ethyl acetate extract obtained from the glyceric extract of the investigated propolis by GC-MS after silylation (TMS derivatives).

Compound ^1^	% of TIC ^2^	M^+∙^	Prominent MS Peaks ^3^, *m*/*z*
***Aromatic aldehydes, aromatic acids and their esters***	**1.1**		
Vanillin	0.3	224	209, 194 (100)
Cinnamic acid	0.2	220	205, 161 (100), 145, 131, 103, 75
*p*-Hydroxybenzoic acid	0.1	282	267 (100), 223, 193
*p*-Methoxyphenylacetic acid	0.1	238	223, 208, 193 (100)
Vanillic acid	0.2	312	297, 267, 194, 105 (100), 91, 77
Protocatechuic acid	0.2	370	355 (100), 311
***Diterpenes***	**2.3**		
Pimaric acid	0.4	374	359, 257, 241, 121 (100)
Isopimaric acid	0.8	374	359, 256, 241 (100)
Palustric acid	0.1	374	359, 241 (100)
Dehydroabietic acid	0.4	372	357, 239 (100)
Abietic acid	0.4	374	256 (100), 241, 185
Mercusic (junicedric) acid	0.2	480	465, 362 (100), 121
***Cardanols***	**1.0**		
Cardanol C_17_H_31_	0.1	400	180 (100)
Cardanol C_17_H_35_	0.7	404	180 (100)
Cardanol C_19_H_35_	0.2	430	180 (100)
***Cardols***	**3.7**		
Cardol C_15_H_31_	0.4	464	268 (100)
Cardol C_17_H_31_	1.2	488	268 (100)
Cardol C_17_H_33_	0.1	490	268 (100)
Cardol C_17_H_33_ (isomer)	1.1	490	268 (100)
Cardol C_17_H_35_	0.3	492	268 (100)
Cardol C_19_H_35_	0.6	518	268 (100)
***Anacardic acids***	**2.0**		
Anacardic acid C_15_H_31_	0.3	492	477 (100), 219
Anacardic acid C_17_H_31_	0.5	516	501, 219 (100)
Anacardic acid C_17_H_35_	0.7	520	505 (100), 219
Anacardic acid C_17_H_33_	0.5	546	531 (100), 219
***Lignans***	**3.9**		
Pinoresinol	3.9	502	487, 235, 223 (100), 209
***Triterpenes***	**22.4**		
Lanosterol	0.6	498	483, 393 (100), 109
*β*-Amyrin	2.1	498	218 (100), 203, 189
*α*-Amyrenone	3.0	424	218 (100), 203, 189
*α*-Amyrin	2.1	498	218 (100), 203, 189
Cycloartenol	7.9	498	408 (100), 393, 365, 339
Ursolic acid	1.7	600	482, 320, 203 (100), 189, 133
Oleanolic acid	2.6	600	482, 320, 203 (100), 189
Mangiferolic acid	2.4	600	585, 510, 495, 467 (100), 441, 388
**Others**			
Palmitic acid	0.2	328	313, 129, 117 (100)
Oleic acid	0.1	354	339, 129, 117 (100)
Stearic acid	0.1	356	341, 129, 117 (100)
Arachidic acid	0.1	384	369, 129, 117 (100)
3-Hydroxyheneicosenoic acid	0.6	484	469, 427, 233 (100)

^1^ The name given does not include TMS substituents. ^2^ The ion current generated depends on the characteristics of the compound concerned and is not a true quantification. ^3^ (100)—base peak.

## Data Availability

The data are publicly available and will be made available upon request.

## Supplementary Materials

The following supporting information can be downloaded at: https://www.mdpi.com/article/10.3390/nu16121880/s1.
